# Dibromido{*N*′-[1-(pyridin-2-yl)ethyl­idene]picolinohydrazide-κ^2^ *N*′,*O*}cadmium

**DOI:** 10.1107/S1600536812023185

**Published:** 2012-05-31

**Authors:** Mehmet Akkurt, Ali Akbar Khandar, Muhammad Nawaz Tahir, Seyed Abolfazl Hosseini Yazdi, Farhad Akbari Afkhami

**Affiliations:** a Department of Physics, Faculty of Sciences, Erciyes University, 38039 Kayseri, Turkey; b Department of Inorganic Chemistry, Faculty of Chemistry, University of Tabriz, 51666 Tabriz, Iran; c Department of Physics, University of Sargodha, Sargodha, Pakistan

## Abstract

The title compound, [CdBr_2_(C_13_H_12_N_4_O)], was obtained from the reaction of Cd(NO_3_)_2_·4H_2_O with meth­yl(pyridin-2-yl)methanone picolinoylhydrazone and sodium bromide. The Cd^2+^ cation is ligated by one O atom and two N atoms of the tridentate ligand and two bromide anions, forming a Br_2_CdN_2_O polyhedron with a distorted trigonal–bipyramidal coordination geometry. In the crystal, non-classical C—H⋯Br hydrogen bonds are observed. In addition, π–π stacking inter­actions [centroid–centroid distance = 3.7455 (19) Å] contribute to the stabilization of the crystal structure.

## Related literature

For related complexes with similar tridentate ligands, see: Kasuga *et al.* (2001 ▶); Chen *et al.* (2005 ▶); Datta *et al.* (2011 ▶).
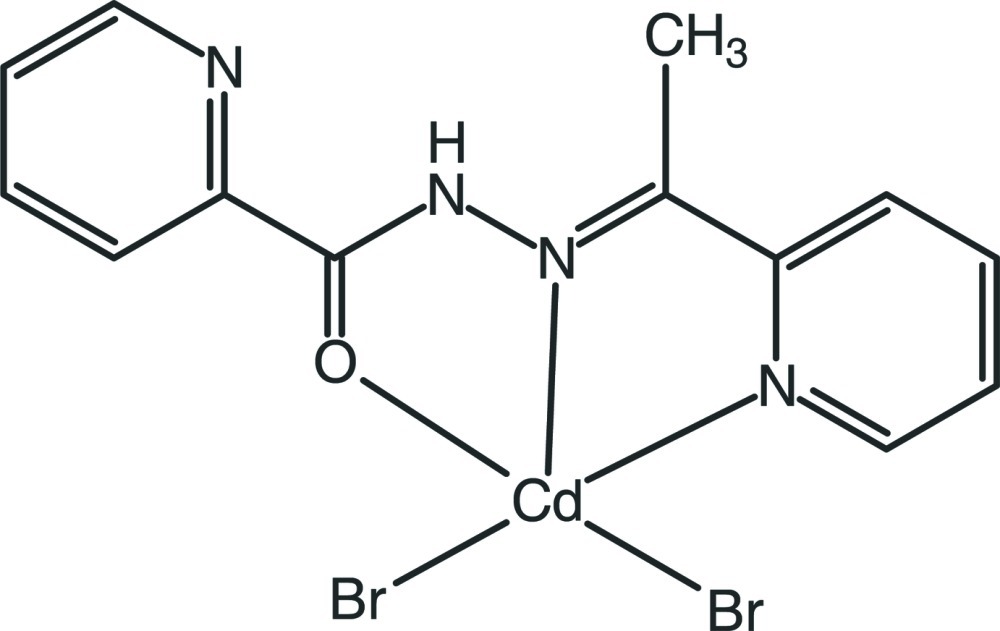


## Experimental

### 

#### Crystal data

[CdBr_2_(C_13_H_12_N_4_O)]
*M* *_r_* = 512.48
Monoclinic, 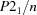

*a* = 8.1336 (3) Å
*b* = 13.6111 (5) Å
*c* = 14.6102 (5) Å
β = 90.550 (1)°
*V* = 1617.38 (10) Å^3^
*Z* = 4
Mo *K*α radiation
μ = 6.29 mm^−1^
*T* = 296 K
0.32 × 0.18 × 0.16 mm

#### Data collection

Bruker Kappa APEXII CCD diffractometer
Absorption correction: multi-scan (*SADABS*; Bruker, 2005 ▶) *T*_min_ = 0.267, *T*_max_ = 0.365
15317 measured reflections
3874 independent reflections
2859 reflections with *I* > 2σ(*I*)
*R*_int_ = 0.032

#### Refinement

*R*[*F* ^2^ > 2σ(*F* ^2^)] = 0.028
*wR*(*F* ^2^) = 0.062
*S* = 1.02
3874 reflections
195 parameters
1 restraint
H atoms treated by a mixture of independent and constrained refinement
Δρ_max_ = 0.47 e Å^−3^
Δρ_min_ = −0.54 e Å^−3^

### 

Data collection: *APEX2* (Bruker, 2009 ▶); cell refinement: *SAINT* (Bruker, 2009 ▶); data reduction: *SAINT*; program(s) used to solve structure: *SHELXS97* (Sheldrick, 2008 ▶); program(s) used to refine structure: *SHELXL97* (Sheldrick, 2008 ▶); molecular graphics: *ORTEP-3 for Windows* (Farrugia, 1997 ▶) and *PLATON* (Spek, 2009 ▶); software used to prepare material for publication: *WinGX* (Farrugia, 1999 ▶) and *PLATON*.

## Supplementary Material

Crystal structure: contains datablock(s) global, I. DOI: 10.1107/S1600536812023185/vm2176sup1.cif

Structure factors: contains datablock(s) I. DOI: 10.1107/S1600536812023185/vm2176Isup2.hkl

Additional supplementary materials:  crystallographic information; 3D view; checkCIF report

## Figures and Tables

**Table 1:** Hydrogen-bond geometry (Å, °)

*D*—H⋯*A*	*D*—H	H⋯*A*	*D*⋯*A*	*D*—H⋯*A*
C7—H7*C*⋯Br2^i^	0.96	2.91	3.810 (4)	157

Symmetry code: (i) 
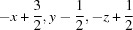
.

**Fig. 1.**
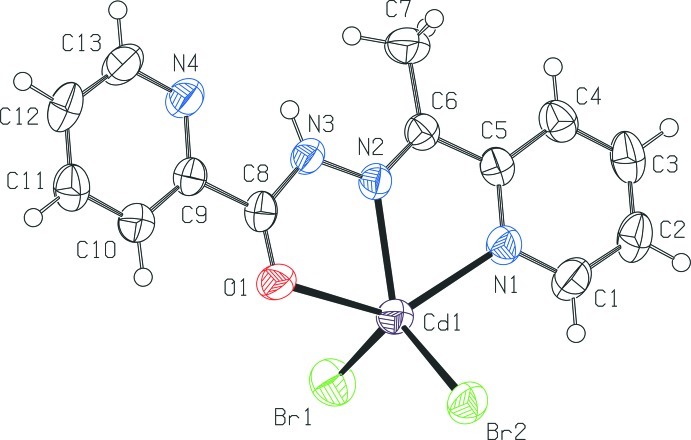
The molecular structure of the title compound with the atom numbering scheme. Displacement ellipsoids for non-H atoms are drawn at the 50% probability level.

**Fig. 2.**
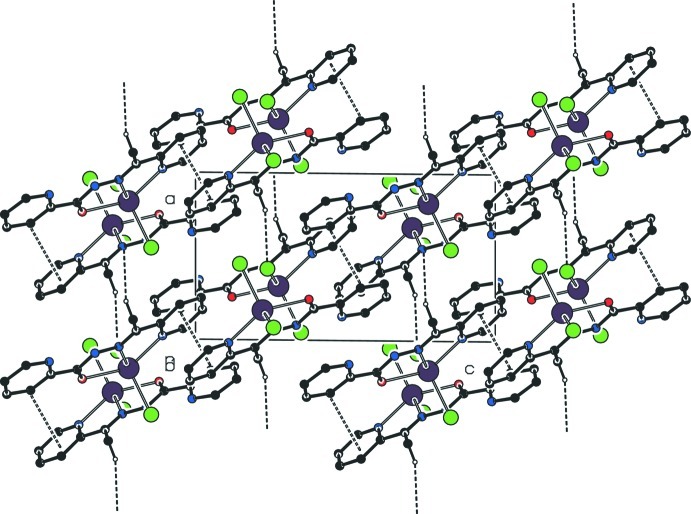
View of the intermolecular C—H···Br hydrogen bonds and π-π stacking interactions of the title compound. H atoms not involved in hydrogen bonding are omitted.

## supplementary crystallographic information

### Comment

Schiff base complexes have attracted much attention due to their interesting structures and wide potential applications. Recently, the relative unsymmetrical tridentate Schiff base ligands and their hydrogenated derivatives have been introduced into the coordination chemistry to assemble polymers with beautiful molecular structures. Some organic N-donor ligands are often chosen to fabricate these various complexes. In this connection, some complexes with similar tridentate ligands have been studied (Kasuga *et al.*, 2001; Chen *et al.*, 2005; Datta *et al.*, 2011). Herein, we report the structure of a new cadmium complex based on a pyridine based tridentate Schiff base ligand.

The molecular structure of title compound is shown in Fig. 1. The Cd ion is five coordinated forming a distorted trigonal-bipyramidal coordination sphere, in which three positions are occupied by two N atoms and one O atom from the tridentate Schiff base ligand, and two positions coming from two bromide ions. As can be seen in Fig. 1, all non-H atoms of the tridentate Schiff base ligand are nearly coplanar, with maximum deviations of -0.053 (4) Å for C7 and 0.049 (2) Å for N2.

Molecules are linked to each other, *via* weak C—H···Br intermolecular hydrogen bonds along the crystallographic *a* axis (Table 1, Fig. 2). In the crystal, weak π-π stacking interactions also contribute to the stabilization: [*Cg*3···*Cg*3(1 - *x*, -*y*, 1 - *z*) = 3.7455 (19) Å; where *Cg*3 is the centroid of the N1/C1–C5 ring].

### Experimental

The potentially tridenatate ligand methyl-2-pyridyl ketone picolinoyl hydrazone was obtained by condensation of methyl-2-pyridyl ketone and picolinic acid hydrazide with the ratio 1:1 in methanol. The title compound C_13_H_12_Br_2_CdN_4_O has been synthesized by the reaction of methanolic solution of the ligand and Cd(NO_3_)_2_.4H_2_O in the presence of excess amount of NaBr. The ligand (1 mmol, 0.240 g) and cadmium nitrate (1 mmol, 279 g) were placed in main arm of a branched tube; sodium bromide (2 mmol, 0.206 g) was added to the mixture too. Methanol was carefully added to fill the arms. The tube was sealed and the ligand-containing arm was immersed in an oil bath at 333 K while the branched arm was kept at ambient temperature. After five days, suitable single crystals, were deposited in the cooler arm which were isolated, filtered off, washed with acetone and ether and air dried.

### Refinement

H atoms bonded to C atoms were positioned geometrically and treated as riding with C—H = 0.93 Å and *U*_iso_(H) = 1.2U_eq_(C) for aromatic H, and C—H = 0.96 Å and *U*_iso_(H) = 1.5U_eq_(C) for methyl H. The amine H atom was located in difference Fourier map and refined freely [N—H = 0.86 (2) Å].

### Crystal data

**Table:**

[CdBr_2_(C_13_H_12_N_4_O)]	*F*(000) = 976
*M**_r_* = 512.48	*D*_x_ = 2.105 Mg m^−^^3^
Monoclinic, *P*2_1_/*n*	Mo *K*α radiation, λ = 0.71073 Å
Hall symbol: -P 2yn	Cell parameters from 545 reflections
*a* = 8.1336 (3) Å	θ = 4.2–18.3°
*b* = 13.6111 (5) Å	µ = 6.29 mm^−^^1^
*c* = 14.6102 (5) Å	*T* = 296 K
β = 90.550 (1)°	Prism, colourless
*V* = 1617.38 (10) Å^3^	0.32 × 0.18 × 0.16 mm
*Z* = 4	

### Data collection

**Table:**

Bruker Kappa APEXII CCD diffractometer	3874 independent reflections
Radiation source: fine-focus sealed tube	2859 reflections with *I* > 2σ(*I*)
Graphite monochromator	*R*_int_ = 0.032
ω scans	θ_max_ = 28.1°, θ_min_ = 2.9°
Absorption correction: multi-scan (*SADABS*; Bruker, 2005)	*h* = −10→10
*T*_min_ = 0.267, *T*_max_ = 0.365	*k* = −17→18
15317 measured reflections	*l* = −19→18

### Refinement

**Table:**

Refinement on *F*^2^	Primary atom site location: structure-invariant direct methods
Least-squares matrix: full	Secondary atom site location: difference Fourier map
*R*[*F*^2^ > 2σ(*F*^2^)] = 0.028	Hydrogen site location: inferred from neighbouring sites
*wR*(*F*^2^) = 0.062	H atoms treated by a mixture of independent and constrained refinement
*S* = 1.02	*w* = 1/[σ^2^(*F*_o_^2^) + (0.0245*P*)^2^ + 0.5132*P*] where *P* = (*F*_o_^2^ + 2*F*_c_^2^)/3
3874 reflections	(Δ/σ)_max_ = 0.001
195 parameters	Δρ_max_ = 0.47 e Å^−^^3^
1 restraint	Δρ_min_ = −0.54 e Å^−^^3^

### Special details

**Table:**

Geometry. Bond distances, angles *etc*. have been calculated using the rounded fractional coordinates. All su's are estimated from the variances of the (full) variance-covariance matrix. The cell e.s.d.'s are taken into account in the estimation of distances, angles and torsion angles
Refinement. Refinement on *F*^2^ for ALL reflections except those flagged by the user for potential systematic errors. Weighted *R*-factors *wR* and all goodnesses of fit *S* are based on *F*^2^, conventional *R*-factors *R* are based on *F*, with *F* set to zero for negative *F*^2^. The observed criterion of *F*^2^ > σ(*F*^2^) is used only for calculating -*R*-factor-obs *etc*. and is not relevant to the choice of reflections for refinement. *R*-factors based on *F*^2^ are statistically about twice as large as those based on *F*, and *R*-factors based on ALL data will be even larger.

### Fractional atomic coordinates and isotropic or equivalent isotropic displacement parameters (Å^2^)

**Table:**

	*x*	*y*	*z*	*U*_iso_*/*U*_eq_	
Cd1	0.32238 (3)	0.16938 (2)	0.27655 (2)	0.0429 (1)	
Br1	0.04606 (5)	0.15378 (3)	0.35202 (3)	0.0626 (1)	
Br2	0.42596 (5)	0.34049 (2)	0.23982 (3)	0.0550 (1)	
O1	0.2555 (3)	0.10824 (15)	0.12175 (14)	0.0493 (8)	
N1	0.5195 (3)	0.12698 (18)	0.38976 (17)	0.0438 (9)	
N2	0.4511 (3)	0.02135 (16)	0.24438 (15)	0.0370 (8)	
N3	0.4106 (3)	−0.02318 (18)	0.16427 (17)	0.0401 (8)	
N4	0.3565 (3)	−0.11450 (19)	0.01147 (18)	0.0495 (10)	
C1	0.5632 (5)	0.1867 (2)	0.4579 (2)	0.0555 (11)	
C2	0.6781 (5)	0.1618 (3)	0.5230 (2)	0.0585 (11)	
C3	0.7498 (4)	0.0712 (3)	0.5186 (2)	0.0585 (14)	
C4	0.7079 (4)	0.0090 (2)	0.4477 (2)	0.0487 (11)	
C5	0.5925 (4)	0.0390 (2)	0.3832 (2)	0.0385 (9)	
C6	0.5464 (3)	−0.0217 (2)	0.3021 (2)	0.0377 (9)	
C7	0.6137 (5)	−0.1226 (2)	0.2915 (2)	0.0577 (11)	
C8	0.3114 (4)	0.0268 (2)	0.1050 (2)	0.0392 (9)	
C9	0.2810 (4)	−0.0274 (2)	0.01743 (19)	0.0381 (9)	
C10	0.1844 (4)	0.0113 (2)	−0.0505 (2)	0.0473 (11)	
C11	0.1592 (4)	−0.0425 (2)	−0.1296 (2)	0.0523 (11)	
C12	0.2361 (5)	−0.1319 (2)	−0.1370 (2)	0.0543 (11)	
C13	0.3333 (5)	−0.1652 (2)	−0.0658 (2)	0.0569 (13)	
H1	0.51340	0.24800	0.46140	0.0660*	
H2	0.70680	0.20560	0.56930	0.0700*	
H3	0.82590	0.05170	0.56290	0.0700*	
H3N	0.434 (4)	−0.0819 (15)	0.150 (2)	0.056 (10)*	
H4	0.75670	−0.05260	0.44310	0.0580*	
H7A	0.54530	−0.15920	0.24990	0.0860*	
H7B	0.61610	−0.15480	0.34990	0.0860*	
H7C	0.72320	−0.11890	0.26780	0.0860*	
H10	0.13630	0.07280	−0.04360	0.0570*	
H11	0.09200	−0.01880	−0.17650	0.0630*	
H12	0.22280	−0.16960	−0.18970	0.0650*	
H13	0.38510	−0.22580	−0.07180	0.0680*	

### Atomic displacement parameters (Å^2^)

**Table:**

	*U*^11^	*U*^22^	*U*^33^	*U*^12^	*U*^13^	*U*^23^
Cd1	0.0474 (1)	0.0383 (1)	0.0428 (1)	0.0045 (1)	−0.0051 (1)	−0.0016 (1)
Br1	0.0532 (2)	0.0726 (3)	0.0622 (2)	−0.0011 (2)	0.0063 (2)	0.0021 (2)
Br2	0.0638 (2)	0.0413 (2)	0.0598 (2)	−0.0049 (2)	−0.0091 (2)	0.0024 (2)
O1	0.0616 (15)	0.0432 (13)	0.0429 (12)	0.0105 (11)	−0.0085 (11)	−0.0083 (10)
N1	0.0503 (16)	0.0397 (14)	0.0412 (15)	−0.0023 (12)	−0.0086 (12)	0.0021 (11)
N2	0.0393 (14)	0.0367 (13)	0.0350 (14)	−0.0022 (11)	−0.0031 (11)	−0.0016 (10)
N3	0.0476 (15)	0.0354 (14)	0.0373 (14)	−0.0005 (12)	−0.0045 (11)	−0.0063 (11)
N4	0.0654 (19)	0.0394 (15)	0.0436 (16)	0.0006 (13)	−0.0089 (13)	−0.0043 (12)
C1	0.075 (2)	0.0403 (18)	0.051 (2)	−0.0047 (17)	−0.0117 (18)	−0.0030 (15)
C2	0.071 (2)	0.061 (2)	0.0433 (19)	−0.0144 (19)	−0.0141 (17)	−0.0057 (16)
C3	0.056 (2)	0.073 (3)	0.046 (2)	−0.0134 (18)	−0.0175 (17)	0.0083 (17)
C4	0.0471 (19)	0.0505 (19)	0.0483 (19)	−0.0019 (15)	−0.0065 (15)	0.0107 (15)
C5	0.0371 (16)	0.0401 (16)	0.0381 (16)	−0.0056 (13)	−0.0025 (13)	0.0074 (13)
C6	0.0359 (16)	0.0361 (16)	0.0411 (17)	−0.0045 (13)	0.0005 (13)	0.0033 (13)
C7	0.065 (2)	0.0458 (19)	0.062 (2)	0.0137 (17)	−0.0072 (18)	−0.0003 (17)
C8	0.0396 (16)	0.0418 (17)	0.0362 (16)	−0.0092 (14)	−0.0016 (13)	0.0004 (13)
C9	0.0384 (16)	0.0383 (16)	0.0376 (16)	−0.0058 (13)	0.0000 (13)	−0.0005 (12)
C10	0.0495 (19)	0.0458 (18)	0.0464 (19)	0.0008 (15)	−0.0065 (15)	−0.0043 (15)
C11	0.056 (2)	0.057 (2)	0.0437 (19)	−0.0102 (17)	−0.0101 (16)	0.0044 (15)
C12	0.069 (2)	0.053 (2)	0.0408 (19)	−0.0194 (18)	−0.0046 (17)	−0.0082 (15)
C13	0.075 (3)	0.0397 (18)	0.056 (2)	−0.0021 (17)	−0.0054 (19)	−0.0107 (16)

### Geometric parameters (Å, º)

**Table:**

Cd1—Br1	2.5218 (5)	C5—C6	1.490 (4)
Cd1—Br2	2.5359 (4)	C6—C7	1.487 (4)
Cd1—O1	2.466 (2)	C8—C9	1.495 (4)
Cd1—N1	2.364 (2)	C9—C10	1.366 (4)
Cd1—N2	2.321 (2)	C10—C11	1.382 (4)
O1—C8	1.224 (4)	C11—C12	1.373 (4)
N1—C1	1.331 (4)	C12—C13	1.377 (5)
N1—C5	1.341 (4)	C1—H1	0.9300
N2—N3	1.356 (3)	C2—H2	0.9300
N2—C6	1.282 (4)	C3—H3	0.9300
N3—C8	1.360 (4)	C4—H4	0.9300
N4—C9	1.338 (4)	C7—H7A	0.9600
N4—C13	1.335 (4)	C7—H7B	0.9600
N3—H3N	0.85 (2)	C7—H7C	0.9600
C1—C2	1.370 (5)	C10—H10	0.9300
C2—C3	1.366 (6)	C11—H11	0.9300
C3—C4	1.378 (4)	C12—H12	0.9300
C4—C5	1.385 (4)	C13—H13	0.9300
			
Br1—Cd1—Br2	117.98 (2)	O1—C8—N3	123.0 (3)
Br1—Cd1—O1	100.54 (6)	O1—C8—C9	124.0 (3)
Br1—Cd1—N1	105.95 (6)	C8—C9—C10	121.4 (3)
Br1—Cd1—N2	114.91 (6)	N4—C9—C8	114.9 (3)
Br2—Cd1—O1	100.76 (5)	N4—C9—C10	123.8 (3)
Br2—Cd1—N1	98.50 (6)	C9—C10—C11	118.9 (3)
Br2—Cd1—N2	127.09 (6)	C10—C11—C12	118.1 (3)
O1—Cd1—N1	134.55 (8)	C11—C12—C13	119.4 (3)
O1—Cd1—N2	67.49 (8)	N4—C13—C12	123.0 (3)
N1—Cd1—N2	68.01 (8)	N1—C1—H1	118.00
Cd1—O1—C8	114.21 (19)	C2—C1—H1	119.00
Cd1—N1—C1	123.3 (2)	C1—C2—H2	121.00
Cd1—N1—C5	117.77 (19)	C3—C2—H2	121.00
C1—N1—C5	118.9 (3)	C2—C3—H3	120.00
Cd1—N2—N3	117.17 (17)	C4—C3—H3	120.00
Cd1—N2—C6	122.33 (18)	C3—C4—H4	120.00
N3—N2—C6	120.3 (2)	C5—C4—H4	120.00
N2—N3—C8	117.6 (2)	C6—C7—H7A	109.00
C9—N4—C13	116.9 (3)	C6—C7—H7B	109.00
C8—N3—H3N	117 (2)	C6—C7—H7C	109.00
N2—N3—H3N	126 (2)	H7A—C7—H7B	109.00
N1—C1—C2	123.0 (3)	H7A—C7—H7C	109.00
C1—C2—C3	118.7 (3)	H7B—C7—H7C	109.00
C2—C3—C4	119.2 (3)	C9—C10—H10	121.00
C3—C4—C5	119.4 (3)	C11—C10—H10	121.00
N1—C5—C4	120.8 (3)	C10—C11—H11	121.00
C4—C5—C6	122.8 (3)	C12—C11—H11	121.00
N1—C5—C6	116.4 (3)	C11—C12—H12	120.00
N2—C6—C7	125.1 (3)	C13—C12—H12	120.00
C5—C6—C7	120.3 (2)	N4—C13—H13	119.00
N2—C6—C5	114.6 (2)	C12—C13—H13	119.00
N3—C8—C9	113.0 (2)		
			
Br1—Cd1—O1—C8	−107.6 (2)	Cd1—N2—C6—C7	170.9 (2)
Br2—Cd1—O1—C8	131.0 (2)	Cd1—N2—N3—C8	6.6 (3)
N1—Cd1—O1—C8	17.6 (3)	Cd1—N2—C6—C5	−11.0 (3)
N2—Cd1—O1—C8	5.2 (2)	N3—N2—C6—C7	−3.4 (4)
Br1—Cd1—N1—C1	−76.1 (3)	N3—N2—C6—C5	174.8 (2)
Br2—Cd1—N1—C1	46.4 (3)	N2—N3—C8—O1	−1.5 (4)
O1—Cd1—N1—C1	160.7 (2)	N2—N3—C8—C9	177.5 (2)
N2—Cd1—N1—C1	173.0 (3)	C9—N4—C13—C12	−0.5 (5)
Br1—Cd1—N1—C5	105.4 (2)	C13—N4—C9—C8	−179.7 (3)
Br2—Cd1—N1—C5	−132.2 (2)	C13—N4—C9—C10	−0.4 (5)
O1—Cd1—N1—C5	−17.9 (3)	N1—C1—C2—C3	−0.8 (6)
N2—Cd1—N1—C5	−5.6 (2)	C1—C2—C3—C4	1.8 (5)
Br1—Cd1—N2—N3	85.55 (18)	C2—C3—C4—C5	−0.9 (5)
Br2—Cd1—N2—N3	−92.50 (19)	C3—C4—C5—N1	−1.0 (5)
O1—Cd1—N2—N3	−5.94 (17)	C3—C4—C5—C6	177.0 (3)
N1—Cd1—N2—N3	−176.5 (2)	N1—C5—C6—N2	5.2 (4)
Br1—Cd1—N2—C6	−88.8 (2)	C4—C5—C6—C7	5.3 (4)
Br2—Cd1—N2—C6	93.1 (2)	C4—C5—C6—N2	−172.9 (3)
O1—Cd1—N2—C6	179.7 (2)	N1—C5—C6—C7	−176.6 (3)
N1—Cd1—N2—C6	9.2 (2)	O1—C8—C9—N4	178.4 (3)
Cd1—O1—C8—C9	177.1 (2)	N3—C8—C9—C10	−179.9 (3)
Cd1—O1—C8—N3	−4.0 (4)	O1—C8—C9—C10	−1.0 (5)
Cd1—N1—C5—C6	2.6 (3)	N3—C8—C9—N4	−0.6 (4)
Cd1—N1—C1—C2	−179.7 (3)	N4—C9—C10—C11	1.4 (5)
C1—N1—C5—C4	2.0 (4)	C8—C9—C10—C11	−179.3 (3)
C1—N1—C5—C6	−176.1 (3)	C9—C10—C11—C12	−1.5 (5)
C5—N1—C1—C2	−1.1 (5)	C10—C11—C12—C13	0.7 (5)
Cd1—N1—C5—C4	−179.3 (2)	C11—C12—C13—N4	0.3 (6)
C6—N2—N3—C8	−178.9 (3)		

### Hydrogen-bond geometry (Å, º)

**Table:**

*D*—H···*A*	*D*—H	H···*A*	*D*···*A*	*D*—H···*A*
C7—H7*C*···Br2^i^	0.96	2.91	3.810 (4)	157

Symmetry code: (i) −*x*+3/2, *y*−1/2, −*z*+1/2.
